# Association of mindfulness on state-trait anxiety in choking-susceptible athletes: mediating roles of resilience and perceived stress

**DOI:** 10.3389/fpsyg.2023.1232929

**Published:** 2023-08-29

**Authors:** Yiwei Tang, Longjun Jing, Yang Liu, Huilin Wang

**Affiliations:** ^1^School of Physical Education, Hunan University of Science and Technology, Xiangtan, China; ^2^School of Business, Hunan University of Science and Technology, Xiangtan, China

**Keywords:** choking-susceptible athletes, mindfulness, resilience, perceived stress, state-trait anxiety

## Abstract

**Introduction:**

It is a well-documented psychological phenomenon for athletes to experience abnormal performance on the field, often called choking. Negative emotions such as perceived stress and state-trait anxiety have been linked to this phenomenon. In an effort to delve into the intricate relationship between mindfulness and state-trait anxiety among athletes susceptible to choking, this study was conducted in Central China during the period from October to November 2022.

**Methods:**

The sample selection process employed a combination of cluster sampling and random sampling, resulting in a total of 377 viable samples encompassing choking-susceptible athletes who frequently grapple with state-trait anxiety and demonstrate performance deviations. The data analysis was executed utilizing AMOS v.26.

**Results:**

The results indicate a negative association between mindfulness and perceived stress (standardized coefficient = −0.224, *p* < 0.001), resilience and perceived stress (standardized coefficient = −0.237, *p* < 0.001), as well as perceived stress and state-trait anxiety (standardized coefficient = 0.510, *p* < 0.001). The positive impact of mindfulness on state-trait anxiety is mediated by resilience and perceived stress (standardized indirect effect = 0.237, *p* < 0.001). The explanatory power of this study is *R*^2^ = 0.35.

**Discussion:**

Drawing from these substantial findings, a key recommendation is to implement mindfulness training programs for athletes prone to choking. This proactive measure, facilitated by sports departments, clubs, and coaches, could potentially alleviate state-trait anxiety, enhancing athletes’ mental well-being and optimizing their performance outcomes during competitions.

## 1. Introduction

In high-stakes sports competitions, athletes are expected to perform at their best under pressure. However, some athletes may experience a phenomenon called choking, which leads to abnormal performance and can result in a loss. Many sports psychology scholars have defined choking as the result of athletes trying too hard to win the championship (Baumeister, 1984; Hill et al., 2010). Pressure is believed to destroy habitual exercise methods, leading to a decline in performance and reflecting the participation of effort (Baumeister and Showers, 1986). Although factors such as pre-match preparations, physical conditions, and injuries can also contribute to poor performance, choking is common among high-level athletes, especially during critical stages of the game. Research has shown that, even with high motivation and subjective efforts to perform well, athletes who perceive pressure often exhibit lower than expected or usual performance, making choking a cognitive to execution process (Papathomas, 2007). It is primarily influenced by the combined effects of stable factors, such as self-awareness and anxiety characteristics, and unstable factors, such as internal and external incentives (Krane et al., 1995; Anshel, 2011). In addition, some scholars believe stress can cause athletes to lose control and negatively affect their performance (Wang et al., 2004). The choking phenomenon illustrates that success in high-level competitions requires a high level of physical ability, technique, tactics, and sound psychological qualities.

Athletes commonly experience sports anxiety, which can hurt their performance, causing them to underperform in critical moments. State-trait anxiety refers to a temporary emotional state of restlessness, including tension and fear, which perceives the movement situation as a threat. It comprises three components: state self-confidence, cognitive anxiety, and physical anxiety (Wilson et al., 2009). According to Spielberger (1983), state-trait anxiety is a short-term emotional state triggered by the perception of dangerous stimuli, such as personal tension, worry, anxiety, distress, and overexcitement of the autonomic nervous system. The leading control theory of exercise suggests that stress-induced anxiety is associated with cognitive bias (Eysenck and Wilson, 2016). Research has demonstrated that anxious individuals are more inclined to focus on threatening stimuli and interpret stress as an unfavorable factor for performance (Bar-Haim et al., 2007). Additionally, the study found that cognitive bias is crucial in whether anxiety occurs under pressure. Athletes who interpret stress or competitive situations as threatening are more likely to develop anxiety, affecting sports performance (Eysenck and Wilson, 2016). In light of these insights, the choice to target state-trait anxiety as the focal outcome variable in this study attains a heightened level of specificity, effectively honing in on the performance dynamics of athletes vulnerable to choking in high-stakes competitive environments.

Simultaneously, in athletes, personal and environmental factors influence their emotions before and after competitions, leading to psychological changes, with perceived stress and trait anxiety being common emotional issues. Given the positive correlation between perceived stress and psychological and health-related problems, there is a causal relationship between perceived stress and trait anxiety. Many scholars have verified the relationship between perceived stress and trait anxiety. For example, Ghorbani et al. (2008) found that individuals with high levels of perceived stress often experience higher levels of anxiety and depression, regardless of their race and cultural characteristics. Similarly, Bedini et al. (2011) reported a negative correlation between perceived stress and indicators of quality of life. Perceived stress can impact an individual’s life and potentially lead to depression and anxiety. For athletes, those with higher levels of perceived stress often exhibit poorer emotional and subjective performance during competitions, leading to anxiety (Nicholls et al., 2012). The above-mentioned research demonstrates the direct or indirect effects of perceived stress on anxiety. Secondly, resilience, as a positive coping behavior, has been shown in the literature to help individuals effectively cope with stress (Aspinwall and Taylor, 1997). For example, Lehrer et al. (2020) found that individuals with resilience are unlikely to experience negative emotions as they are more proactive in managing the associated psychological stress. Similarly, if individuals are facing various mental health problems such as loneliness, anxiety, depression, or insomnia, those with lower resilience are expected to experience higher levels of stress, making them more prone to anxiety (Tudose, 2021). Thirdly, existing literature indicates that mindfulness and resilience significantly and directly affect individuals’ perceived stress, indirectly explaining lower levels of trait anxiety. Lemay et al. (2021) found a negative correlation between mindfulness and stress and anxiety levels. Therefore, in many previous studies, mindfulness directly influenced resilience, perceived stress, and anxiety, or mediated anxiety through variables such as fatigue and self-efficacy. Given the correlation between resilience and perceived stress and the impact of mindfulness on trait anxiety, it is reasonable to hypothesize a relationship among these factors. This study attempts to explore an important and indirect pathway from mindfulness to trait anxiety, examining how resilience and perceived stress influence trait anxiety.

Since the 1980s, researchers in sports psychology have been interested in athletes’ abnormal performance during competitions, known as choking (Hill et al., 2010). Considerable progress has been made in understanding the mechanism underlying this phenomenon. Researchers have explored various perspectives, including interference theory and self-monitoring theory, to explain how attention shifting can lead to choking. Wang (2002) proposed a comprehensive choking process theory that considers task characteristics, skill levels, coping strategies, and the impact of individual factors on choking. However, the relationship between the different factors that induce athletes’ choking needs further study. Stress is a critical factor that can induce abnormal performance in athletes, and individuals with solid self-awareness may be more vulnerable to external stimuli (Geukes, 2013). The ultimate goal of this research is to develop effective interventions to prevent choking in athletes. Current interventions, such as mindfulness training, dual-task intervention (Wulf, 2013), and music therapy (Mesagno et al., 2009), have shown some promise in preventing choking by activating different brain areas. Nevertheless, the existing body of research concerning mindfulness interventions specifically targeting athletes facing choking episodes remains somewhat scant, necessitating additional validation of their efficacy. Given these prevailing limitations, the present study seeks to address a comprehensive set of objectives: (1) to understand the association among various psychological factors that induce athletes’ choking; (2) to examine the impact of mindfulness on athletes’ state-trait anxiety and validate its effectiveness; (3) to investigate the relationship among athletes’ mindfulness, resilience, perceived stress, and state-trait anxiety; (4) to provide recommendations for preventing athletes’ choking phenomenon.

Numerous studies have demonstrated the benefits of mindfulness (Kiken et al., 2017), particularly in reducing stress and anxiety in individuals (Lindsay and Elsie, 2016; Tang et al., 2022). While mindfulness has been explored extensively in psychology literature (Sharma et al., 2014), it has also been applied in organizational research as a trait that helps employees improve job performance and mental health (Charoensukmongkol, 2014). However, its role in abnormal performance in athletes has yet to be extensively studied. This study aims to investigate the association of mindfulness on athletes’ state-trait anxiety, perceived stress, and choking, as these factors are crucial contributors to athletes’ choking phenomenon. However, it remains unclear whether mindfulness can influence athletes’ resilience, perceived stress, and subsequently their state-trait anxiety. This study makes the following contributions: Firstly, this study focuses on choking-susceptible athletes’ stress and trait anxiety intervention, using mindfulness interventions to enhance mental resilience and reduce perceived stress and trait anxiety. This expands the research on the mechanisms through which mindfulness affects athletes’ state-trait anxiety. Additionally, the study explores the mediating effects of resilience and perceived stress on the relationship between mindfulness and trait anxiety to prevent choking in athletes. The results suggest that mindfulness interventions lead to improved resilience and reduced perceived stress and trait anxiety, thereby reducing the incidence of choking in athletes. It highlights the positive resilience developed by choking-susceptible athletes through mindfulness training, enabling them to establish more adaptive perceptions of stress and state anxiety. Moreover, the study extends the research on the micro-level mechanisms of resilience. While previous studies typically treat resilience as the outcome variable, in this study, it serves as a mediating variable. Therefore, this study analyzes the association of mindfulness on choking-susceptible athletes’ perceived and trait anxiety, improves resilience, and reduces blocking when athletes participate in significant competitions. These findings provide effective intervention methods and mean to prevent choking in athletes. In conclusion, this study contributes to a better understanding of choking in athletes, supports previous research, and provides a reference for future research.

The remaining sections of this study are organized as follows: Section 2 provides a comprehensive literature review, which includes relevant theories and hypotheses proposed in this study. Additionally, a conceptual model is presented. Section 3 introduces the data collection, questionnaire composition, and analysis methods. The results of the data analysis and hypothesis testing are presented in Section 4. Section 5 discusses the results and shows the theoretical and practical implications of the study. In Section 6, the limitations of the study are acknowledged and discussed, providing insights into areas for improvement and further research. Finally, Section 7 summarizes the study’s central ideas and suggestions for future research.

## 2. Hypotheses development

### 2.1. Mindfulness, resilience, perceived stress, and state-trait anxiety

As the study of mindfulness has progressed, its definition has gained greater precision. Brown and Ryan (2003) characterize mindfulness as “paying attention to and being aware of what is happening in the present.” The application of mindfulness across diverse domains has also undergone extensive examination (Chiesa and Serretti, 2010). Numerous investigations have underscored the affirmative outcomes of mindfulness in mitigating individuals’ psychological stress and bolstering their physical well-being. Firstly, within many professional contexts, individuals who integrate mindfulness into their routine exhibit enhanced competence in managing external stressors. Illustratively, mindfulness training has proven effective in mitigating work-related stress and ameliorating the overall quality of life for healthcare practitioners (Burton et al., 2017). Secondly, mindfulness empowers individuals to adeptly navigate unwelcome emotional states by preventing excessive attachment or detachment from their emotional experiences (Feldman et al., 2007; Baer et al., 2008). Furthermore, the study by Gal et al. (2021) sought to ascertain the impact of mindfulness interventions on individuals’ well-being and mental health. The study’s findings indicate that such interventions can significantly diminish individuals’ perceived stress, anxiety, and depression.

According to previous research, Cohen et al. (1983) posited stress as a universally recognized perception. Among the most prevalent emotions linked to stress are depression, tension, and anxiety, with perceived stress often manifesting a positive correlation with psychological and health-related challenges. For instance, Kulsoom and Afsar (2015) assessed the psychological well-being of medical students and discerned an association between elevated stress levels and heightened anxiety. Similarly, Lebares et al. (2018) identified a correlation between elevated burnout levels, intense stress, and adverse impacts such as depression and anxiety among surgical residents in the United States. Their study also revealed that mindfulness training and bolstering resilience among medical professionals can mitigate negative emotional states. When individuals across diverse vocations encounter stress, they deploy various psychological interventions to sustain a positive mindset and continue their professional endeavors. Mindfulness stands out as a pivotal intervention method. A plethora of researchers have concurred that mindfulness constitutes a pivotal factor in advancing individuals’ psychological well-being, augmenting cognitive and other capabilities, and mitigating the influence of perceived stress on mental health. Consequently, individuals characterized by heightened levels of mindfulness often exhibit diminished negative emotional responses and reduced stress levels.

Empirical research underscores resilience as a psychological construct encapsulating an individual’s adeptness in adapting to adversity, trauma, or stress (White et al., 2008). This intricate psychological framework encompasses personality attributes such as self-assurance, concentration, emotional regulation, positive cognitive patterns, tenacity, and the capacity for psychological recovery post-trauma (Butt et al., 2010). Furthermore, heightened levels of resilience correlate with amplified positive emotions and enhanced emotional intelligence, facilitating more effective management of perceived stress (Wu et al., 2022).

On one facet, stress is not a static phenomenon but rather an evolving process. While a commonplace experience in daily life, poorly managed stress can precipitate negative emotional states, including depression and anxiety (Ystgaard et al., 1999). Research findings highlight the critical importance of resilience for choking-susceptible athletes. Jones et al. (2002) assert that resilience consistently emerges as a linchpin in the accomplishments of athletes in studies pertaining to high-achieving athletes. Moreover, Jones et al. (2002) posit that resilience, whether an innate genetic predisposition or an acquired psychological advantage, equips athletes to navigate competitions, training regimens, and life’s stressors more effectively than their counterparts. In a similar vein, Madigan and Nicholls (2017) posit that mentally resilient athletes exhibit positive perceptions of external stimuli under pressure, skillful emotional regulation, and optimistic attitudes toward achieving their goals. Consequently, heightened resilience empowers individuals to deftly marshal resources and confront diverse stressors, indicative of its multifaceted nature (Gucciardi et al., 2009). As a protective factor, resilience serves to mitigate the detrimental impact of perceived stress (Thurston et al., 2018), implying that individuals with elevated resilience often experience diminished levels of perceived stress.

Conversely, state-trait anxiety encapsulates emotional responses like unease, restlessness, and apprehension when individuals perceive a challenge in completing tasks. Recent investigations by Trigueros et al. (2020) delve into the interplay of motivation, resilience, and anxiety within volleyball players, revealing a negative correlation between resilience and anxiety to some extent. Similarly, in a case study involving 200 postdoctoral researchers, Gloria and Steinhardt (2016) establish that resilience serves to curtail the likelihood of encountering anxiety or depressive symptoms during postdoctoral training. Furthermore, Scelzo et al. (2018) employ qualitative and quantitative methodologies on older participants, uncovering a positive link between resilience and mental health, while revealing a negative correlation with anxiety. This underscores the dual influence of resilience, not only positively affecting perceived stress but also exerting a favorable impact on anxiety.

The literature converges to demonstrate the interrelation and reciprocal influence between anxiety and stress. Wathelet et al. (2020) investigate the mental well-being of French university students amid the COVID-19 pandemic, unearthing widespread elevated levels of perceived stress, correlating with mental health disorders including severe depression and heightened anxiety. Moreover, research identifies a correlation between athletes’ perceived stress and trait anxiety in the context of paralympic competitions (Belinchon-deMiguel et al., 2019), thus reasonably inferring that athletes’ elevated perceived stress during competitions can precipitate state-trait anxiety. Based on these findings, this study proposes the following four hypotheses.

**Figure 1:**
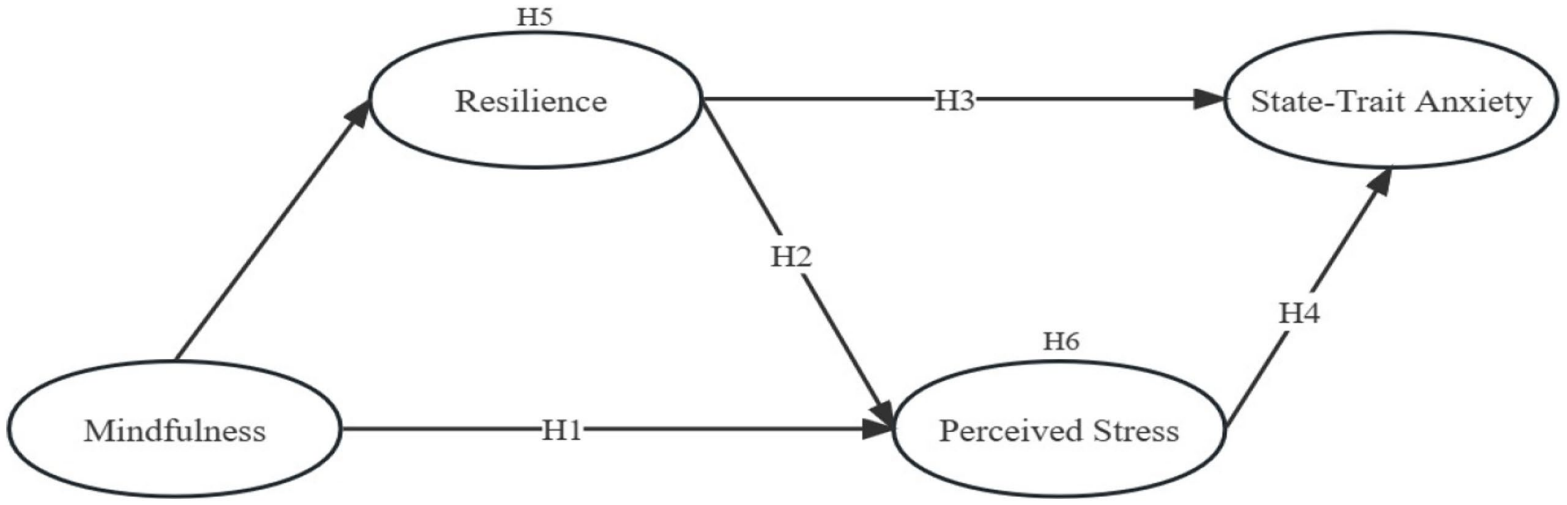
The hypothesized model.

The hypothesized model is shown in Figure 1:

*Hypothesis 1 (H1)*. Mindfulness is negatively related to perceived stress.

*Hypothesis 2 (H2)*. Resilience is negatively related to perceived stress.

*Hypothesis 3 (H3)*. Resilience is negatively related to state-trait anxiety.

*Hypothesis 4 (H4)*. Perceived stress is positively related to state-trait anxiety.

### 2.2. The mediating effects

According to Brown and Ryan (2003), mindfulness is being aware and paying attention to the present moment, being able to maintain a positive state of mind. At the same time, resilience is a positive psychological trait that allows individuals to recover from adversity, uncertainty, conflict, and failure, leading to positive changes, progress, and increased sense of responsibility (Luthans, 2002). Consequently, both mindfulness and resilience hold the potential to positively shape individuals’ emotional responses and facilitate their capacity to confront and adapt to challenging circumstances (Hao et al., 2015). Substantiation for this notion emerges from scholarly research. For instance, Mitchell (2021) underscores that nurturing mindfulness via acceptance and focused attention plays a pivotal role in enhancing the adaptability of nurses during their training. In a similar vein, Masrour et al. (2017) demonstrate that engaging in mindfulness practices can fortify the resilience of couples facing infertility, concurrently ameliorating symptoms of depression, anxiety, and stress. Notably, mindfulness-based intervention initiatives have demonstrated efficacy in alleviating the burdens faced by family caregivers, thereby enhancing their adaptability. This underpins a positive correlation between mindfulness and resilience.

Furthermore, precedent studies have highlighted the potential of mindfulness to ameliorate stress and anxiety (Burton et al., 2017). Some investigations have suggested that resilience may modulate the impact of mindfulness on perceived stress. Consequently, the central focus of this study resides in probing the potential significant interconnections between mindfulness, resilience, perceived stress, and state-trait anxiety, prompting the need for a comprehensive exploration.

In conclusion, previous studies have demonstrated a positive effect of mindfulness on perceived stress in individuals. Additionally, a significant relationship between resilience and perceived stress has been reported. Therefore, it is plausible that resilience partially mediates the effect of mindfulness on perceived stress. Furthermore, perceived stress is significantly related to state-trait anxiety. Therefore, considering the interrelationship between mindfulness, resilience, perceived stress, and state-trait anxiety, it is worth exploring whether mindfulness can mediate it through resilience and perceived stress. Based on this, this study proposes the following two hypotheses:

*Hypothesis 5 (H5)*. Resilience mediates the relationship between mindfulness and perceived stress.

*Hypothesis 6 (H6)*. Resilience and perceived stress mediate the relationship between mindfulness and state-trait anxiety.

## 3. Methodology

### 3.1. Participants and procedure

This study employed cluster and random sampling methods to select participants from among choking-susceptible athletes competing in the provincial games in a province in central China. Participants all self-reported themselves as choking-susceptible athletes due to various reasons. Before the survey, the ethics committee at the first author’s institution approved the research protocol. Then, from October to November 2022, the researchers administered a survey to the participating athletes, who were fully informed about the survey’s objectives and provided informed consent before completing the questionnaire. A total of 400 questionnaires were distributed, out of which 377 valid questionnaires were collected, yielding an effective questionnaire recovery rate of 94.25%.

Table 1 displays the demographic characteristics of the 377 participating athletes who completed the survey. The results indicate that: (1) approximately half of the respondents were aged 18 to 25; (2) slightly more male athletes (54.6%) than female athletes (45.4%) took part in the study; (3) over half of the athletes (57.6%) were college students; (4) regarding sports level, nearly half of the athletes (50.1%) reached the athlete level, while only a tiny proportion (4.5%) achieved the national master level; (5) the sports disciplines included ball sports, track and field, and other types of sports. Among them, ball sports, including basketball, soccer, volleyball, badminton, table tennis, and tennis, accounted for the largest portion with 178 individuals, representing 47.2% of the sample. Meanwhile, track and field events, encompassing sprinting, middle-distance running, long-distance running, relay races, hurdles, long jump, and high jump, accounted for 107 participants, or 28.4% of the total. Other sports, such as cheerleading, swimming, taekwondo, and martial arts, constituted the third category, representing 92 individuals or 24.4% of the cohort. Notably, ball sports dominated the majority of the sample’s engagement.

**Table 1:** Participant profile (*N* = 377).

Profiles	Survey (%)
*Age*	
18–25	216 (57.3%)
26–35	140 (37.1%)
35≤	21 (5.6%)
*Gender*	
Male	206 (54.6%)
Female	171 (45.4%)
*Education level*	
Below high school	28 (7.4%)
High school/vocational school	132 (35%)
College and above	217 (57.6%)
*Sport level*	
No sports grade certificate^a^	188 (49.9%)
Second-level athlete^b^	122 (32.4%)
Tier 1 athlete^c^	50 (13.2%)
National athlete level^d^	17 (4.5%)
*Sports items*	
Ball sports	178 (47.2%)
Track and field	107 (28.4%)
Other sports	92 (24.4%)

a Refers to individuals who did not meet the established performance criteria in the previously mentioned competitions.

b Pertains to participants who successfully meet the performance criteria in championships or championship matches that are co-organized by provincial, regional, or municipal sports administrative departments and education administrative departments.

c Denotes those who satisfy the performance criteria in national sports competitions and encompassing comprehensive sports events, as well as championships or championship matches orchestrated by provincial, regional, or municipal sports administrative departments.

d Signifies participants who fulfill the performance criteria in national-level sports competitions, which include the National Games, National Youth Games, National Championships, National Youth Championships, National Junior Championships, National Indoor Championships, National Grand Prix, National Individual Events, National Team Events, in addition to the National Student Games and National Middle/High School Championships.

The survey questionnaire used in this study was composed of five parts, totaling 23 items. The first part of the questionnaire aimed to gather demographic information from the participants, including age, gender, education level, athletic level, and sport. The second part used the five items from the mindfulness scale developed by Carlson and Brown (2005). An example item from the sample included: “It seems I am ‘running on automatic’, without much awareness of what I am doing.” The third part collected data on resilience, measured using the six items from the mental toughness scale developed by Connor and Davidson (2003). An example item from the sample included: “When things look hopeless, I do not give up.” The fourth part gathered data on perceived stress, calculated using the six items from the perceived stress scale developed by Cohen et al. (1983). An example item from the sample included: “In the last month, how often have you felt nervous and stressed?” Finally, the fifth part collected data on state-trait anxiety using the six items from the scale developed by Spielberger (1983). An example item from the sample included: “Cannot get thoughts out of mind.” All four scales were measured using a five-point Likert scale, ranging from 1 (strongly disagree, never) to 5 (strongly agree, always).

The researchers modified some items in the original scales to ensure that the scales used in the study were appropriate for the Chinese cultural context and research field. A pilot test was then conducted on a sample of high-level collegiate athletes in Changsha, China, to ensure the reliability of the revised scales (Fornell and Larcker, 1981). The pilot test received 75 valid responses, and the results showed that the Cronbach coefficients for all the scales were higher than 0.9, which justified the researchers’ modifications to the scales.

### 3.2. Data analysis

In this study, a structural equation model (SEM) was constructed using AMOS v.26 to examine how choking-susceptible athletes can alleviate state-trait anxiety through mindfulness, with the parameters of the model estimated using a maximum likelihood (ML) estimation method. A two-step modeling approach was employed to evaluate the measurement and structural models (Anderson and Gerbing, 1988). Specifically, the reliability and validity of the model were first comprehensively assessed, followed by an examination of the fit and path coefficients of the hypothesized models, as well as testing for the presence of mediating effects.

We conducted two tests to address common method variance (CMV) issues. First, we performed Harman’s univariate test, which showed that the percentage of variance extracted from the univariate test was 44.01%, below the classical threshold of 50%. This suggests that CMV was not a significant issue in this study (Podsakoff et al., 2012). Second, we adopted the CFA single-factor and two-factor comparison methods proposed by Mossholder et al. (1998). The chi-square value for the univariate model was 5190.6 with 230 degrees of freedom, while the chi-square value for the multivariate model was 239.7 with 224 degrees of freedom. The ratio of the difference in chi-square values to the difference in degrees of freedom for the two models is 825.2, indicating a significant difference between the two models and further supporting the absence of CMV. Hence, we concluded that the impact of CMV on this study is minimal and does not require correction.

## 4. Results

### 4.1. Assessment of the measurement model reliability and validity

This study assessed the reliability and discriminant validity by calculating Cronbach’s α and composite reliability (CR) coefficients for the latent variables (Fornell and Larcker, 1981). All variables had Cronbach’s α coefficients ranging from 0.937 to 0.957. Concurrently, the CR values for each variable demonstrated high reliability, falling within the range of 0.938 to 0.957, surpassing the recommended threshold of 0.9, thus affirming the strong reliability of the measurement model. In addition to this, the average variance extraction (AVE) values exhibited noteworthy results, ranging from 0.715 to 0.814 for all variables. These findings solidify the convergent validity of the study’s measurements, reinforcing the coherence of the underlying constructs. Furthermore, Table 2 shows that all correlation coefficients were smaller than the square root of AVE.

**Table 2:** Discriminant validity test.

Construct	MIN	RE	PS	STA
MIN	**(0.902)**			
RE	0.475^**^	**(0.888)**		
PS	−0.341^**^	−0.332^**^	**(0.845)**	
STA	−0.355^**^	−0.335^**^	0.541^**^	**(0.872)**

The square root of the average variance extracted (AVE) is in the diagonals (bold); off diagonals is a Pearson’s correlation of contracts. **p < 0.01, the following significance standard is the same.

### 4.2. Hypotheses testing results

**Figure 2:**
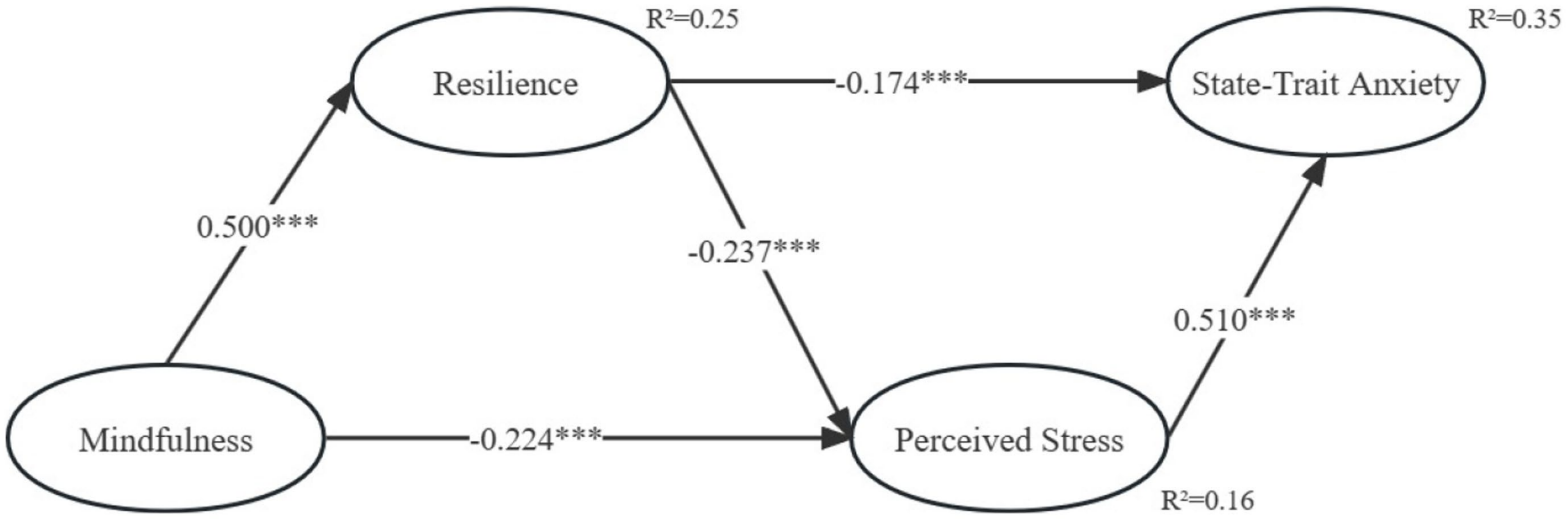
Structural path model. ^***^p < 0.001. Standardized coefficients are reported.

Several tests were conducted to assess the validity and reliability of the structural equation model used in this study. First, the error and residual terms did not show negative values, indicating that the model’s estimates were not violated. Second, the goodness of fit was high (*χ*^2^/*df* = 1.104, GFI = 0.947, AGFI = 0.934, NFI = 0.972, CFI = 0.997, TLI = 0.997, RMSEA = 0.017), indicating that the model fit the data well. Third, the Pearson correlation results in Table 3 showed significant correlations among the independent, mediator, and dependent variables, supporting the hypotheses. Fourth, the results of the structural pathway model in Figure 2 indicated that mindfulness and perceived stress had a significant negative association (*β* = −0.224, *p* < 0.001), supporting H1; resilience and perceived stress had a significant negative association (*β* = −0.237, *p* < 0.001), supporting H2; resilience and state-trait anxiety had a significant negative association (*β* = −0.174, *p* < 0.001), supporting H3; and perceived stress and state-trait anxiety had a significant positive association (*β* = 0.510, *p* < 0.001), supporting H4.

**Table 3:** Indirect effects.

	Point estimate	Product of coefficients		Bootstrapping				
				Percentile 95% CI		Bias-corrected 95% CI		Two-tailed significance
		SE	*Z*	Lower	Upper	Lower	Upper	
MIN → PS	−0.096	0.027	−3.556	−0.174	−0.067	−0.178	−0.071	0.000 (^***^)
MIN → STA	−0.229	0.034	−6.735	−0.329	−0.197	−0.328	−0.196	0.000 (^***^)

Standardized estimations of 5,000 bootstrap samples.

The study hypothesized that mindfulness affects motor state-trait anxiety through two mediators: resilience and perceived stress. To test for mediating effects, the researchers used the bootstrap method (Bollen and Stine, 1990). The results of the 95% confidence intervals of the 5,000 bootstrap samples are shown in Table 3. The absolute values of all *Z* values are significant (greater than 1.96), and the 95% confidence intervals do not include zero. The study found a significant indirect effect between mindfulness and state-trait anxiety via resilience (standardized indirect effect = −0.096, *p* < 0.001), supporting H5. Additionally, resilience and perceived stress significantly mediated the relationship between mindfulness and state-trait anxiety (standardized indirect effect = −0.229, *p* < 0.001), supporting H6. These findings suggest that mindful and resilient athletes are likely to experience lower levels of perceived stress and state-trait anxiety.

## 5. Discussion

### 5.1. Theoretical contribution

This study significantly contributes to the comprehension of the psychological determinants that exert influence on the state-trait anxiety experienced by athletes susceptible to choking. Its primary thrust is to unravel the impact of mindfulness on the state-trait anxiety of these athletes, while concurrently introducing the pivotal roles of resilience and perceived stress as mediating agents. The existing body of research has predominantly focused on the mechanics and catalysts behind choking incidents, yet a dearth of exploration exists regarding the intricate interplay of internal and external factors in conjunction with the psychological underpinnings that culminate in performance anomalies among athletes prone to choking. In this context, the study’s meticulous scrutiny of stress and anxiety, which precipitate choking tendencies, aligns with targeted precision and contributes to the enrichment of pertinent theories. By unraveling the intricate mechanics of athlete choking, this study aspires to pave the way for future research endeavors, enabling the exploration of efficacious intervention modalities. The holistic understanding of choking mechanisms aims to empower athletes and coaches alike with a comprehensive grasp of choking phenomena and avenues for resolution (Beilock and Gray, 2007; Hill et al., 2010).

Extensive investigations have previously established that athletes’ anxiety linked to choking emanates from their perception of external pressures. These external pressures act as catalysts for triggering psychological stress responses, thereby inducing choking occurrences (Wilson et al., 2009). Given the compelling nexus between anxiety, stress, and athletes’ propensity to choke, with heightened state-trait anxiety often translating into suboptimal performance during competitions, this study delves into the imperative of influencing athletes’ perceived stress and state-trait anxiety through mindfulness and resilience interventions. The proposition is substantiated by evidence suggesting that mindfulness, as a potent psychological asset, can mitigate athletes’ anxiety levels, thus ameliorating anomalous performances during competitions (Murray and Janelle, 2003). In consonance, the findings of Medina (2016) which highlight the efficacy of anxiety control in averting athlete choking, align with the present study’s orientation. Consequently, the attenuation of state-trait anxiety could conceivably engender a favorable impact on athletes’ performance, equipping them to attain superior outcomes. Furthermore, this investigation expounds on the correlation between mindfulness and perceived stress, along with the mediating role played by resilience in their dynamic interplay. This insightful exploration adds depth to the understanding of choking-susceptible athletes’ perceived stress, bolstering previous findings put forth by Lemay et al. (2021) and Gal et al. (2021).

In terms of outcomes, the study’s results spotlight mindfulness as the most substantial influencer, with the greatest sway on resilience, followed by perceived stress. Notably, resilience and perceived stress operate as key mediators within the connection linking mindfulness and state-trait anxiety, elucidating a noteworthy 35% of the variance observed in state-trait anxiety, as depicted in Figure 2. The study’s revelations thereby furnish invaluable insights into the intricate dynamic between mindfulness and state-trait anxiety, with a distinct emphasis on the resilience facet among athletes susceptible to choking. By accentuating the pivotal role of resilience, this study proffers a promising avenue for influencing athletes’ state-trait anxiety through mindfulness-based interventions, thereby contributing significantly to both empirical understanding and potential interventions in this domain.

### 5.2. Practical implications

Research has shown that mindfulness can significantly impact the perceived stress and state-trait anxiety of suffocating athletes. The results of this study confirmed the negative correlation between mindfulness and perceived stress, as well as the negative correlation between mindfulness and state-trait anxiety. Moreover, the study also found that psychological resilience and perceived stress mediate the relationship between mindfulness and state-trait anxiety. Considering the various personal and external factors that may cause emotional fluctuations in athletes during competitions, the occurrence of “choking” is prevalent among many athletes. Therefore, the potential adverse effects of mindfulness on athlete performance and mental health are essential considerations.

In light of these insights, it is strongly recommended that coaches and sports organizations prioritize the integration of mindfulness training for athletes susceptible to choking. Such initiatives aim to nurture and enhance athletes’ mindfulness quality. Incorporating mindfulness practices, including meditation, into athletes’ training regimens during periods of rest or prior to and after competitions, holds the potential to facilitate relaxation and efficacious management of performance-related pressures. This proactive approach to mindfulness training can be of immense benefit to all athletes, particularly those prone to choking incidents, given their heightened vulnerability to the influence of performance stress and anxiety. To this end, coaches might consider implementing mandatory mindfulness training as a preemptive measure to curb the likelihood of anxiety manifesting during competitive scenarios, especially for athletes who exhibit heightened emotional susceptibilities.

The importance of mindfulness training is supported by previous research, including Tang et al. (2022), who found a negative correlation between athletes’ mindfulness during competitions and state-trait anxiety, along with its positive impact on athletes’ emotional regulation self-efficacy. Additionally, the views of Lindsay and Elsie (2016) receive further reinforcement. However, despite the proven benefits of mindfulness training, the present landscape of mindfulness initiatives for athletes in domestic settings is less than optimal. This disparity can be attributed to multiple factors, including athletes’ limited awareness of the advantages associated with mindfulness training, coupled with the scarcity of accessible, professionally guided mindfulness programs. This encompasses inadequate infrastructure and a shortage of qualified mental health practitioners well-versed in mindfulness training.

In light of these prevailing challenges, a series of actionable recommendations is put forth. To heighten athletes’ awareness about the pivotal significance of mindfulness training, governmental bodies and sports governing authorities are urged to undertake comprehensive promotional campaigns that spotlight the benefits, techniques, and guidelines underpinning mindfulness practice. Concurrently, sports management entities and coaches should play an active role in motivating athletes to embrace consistent mindfulness training. This necessitates the provision of suitable facilities and a dedicated investment in cultivating a cadre of mental health professionals proficient in mindfulness-based interventions. Through this multifaceted approach, athletes can be empowered with effective coping strategies to curtail state-trait anxiety during competitive engagements, ultimately fostering their holistic well-being and optimized performance outcomes.

## 6. Limitations

This study, while making valuable contributions, is not without its limitations, which warrant careful consideration. Firstly, the study relied on cross-sectional data, which limits the ability to infer causal relationships among the examined variables. Future research should employ longitudinal designs and experimental control groups to provide a more in-depth and comprehensive understanding of these relationships. Secondly, the research model did not consider other moderating variables that may influence the relationships between mindfulness, perceived stress, resilience, and state-trait anxiety. Future studies should take these factors into account to enhance the accuracy and predictive power of the model. Lastly, the study sample targeted choking-susceptible athletes, including a relatively small proportion of national athlete level participants (4.5%), which may limit the generalizability of the findings. Future research should involve a larger number of high-level athletes to validate the results of this study and provide a more comprehensive understanding of the relationship between mindfulness and athletic performance.

## 7. Conclusion

Current cross-sectional survey research indicates that mindfulness has a positive impact on choking-susceptible athletes’ state-trait anxiety. Specifically, this influence of mindfulness on state-trait anxiety is mediated by two variables: resilience and perceived stress. The study findings shed light on the mechanisms through which mindfulness affects choking-susceptible athletes’ state-trait anxiety and contribute to the theoretical understanding of choking-susceptible athletes’ psychological response during times of state-trait anxiety. Considering that choking-susceptible athletes often experience heightened psychological pressure when facing competitive events, it is crucial to encourage coaches to provide psychological training to enhance choking-susceptible athletes’ emotional abilities, ultimately improving their performance in sports.

Furthermore, it is recommended that the National Sports Administration establish relevant training institutions focusing on athletes’ psychological well-being. These institutions can assist athletes representing the country in competitions in addressing psychological pressure and anxiety issues. This will ensure that athletes perform consistently during competitions and reduce the occurrence of choking phenomena.

By incorporating mindfulness-based training and psychological support into athletes’ preparation, athletes can effectively manage their emotions, reduce anxiety, and perform at their best during crucial moments. Establishing such psychological training institutions can be a valuable investment in enhancing athletes’ mental resilience and optimizing their overall athletic performance.

## Data availability statement

The raw data supporting the conclusions of this article will be made available by the authors, without undue reservation.

## Ethics statement

The studies involving human participants were reviewed and approved by the Ethics Committee of the School of Physical Education of Hunan University of Science and Technology (No. ECSPEHUST 2022/0014). The patients/participants provided their written informed consent to participate in this study.

## Author contributions

YT and HW contributed to the conception, design of the study, and organized the database. YT and YL performed the statistical analysis and wrote the first draft of the manuscript. LJ and HW wrote sections of the manuscript. All authors contributed to the article and approved the submitted version.

## Funding

This study was supported by the Hunan Provincial Social Science Committee (No. XSP2023JJZ001) and the Scientific Research Fund of Hunan University of Science and Technology (No. E52203).

## Conflict of interest

The authors declare that the research was conducted in the absence of any commercial or financial relationships that could be construed as a potential conflict of interest.

## Publisher’s note

All claims expressed in this article are solely those of the authors and do not necessarily represent those of their affiliated organizations, or those of the publisher, the editors and the reviewers. Any product that may be evaluated in this article, or claim that may be made by its manufacturer, is not guaranteed or endorsed by the publisher.

## References

[ref1] Anderson J. C. Gerbing D. W. (1988). Structural equation modeling in practice: a review and recommended two-step approach. Psychol. Bull. 103, 411–423. doi: 10.1037//0033-2909.103.3.411

[ref2] Anshel M. H. (2011). Sport psychology: from theory to practice Pearson Benjamin Cummings.

[ref3] Aspinwall L. G. Taylor S. E. (1997). A stitch in time: self-regulation and proactive coping. Psychol. Bull. 121, 417–436. doi: 10.1037//0033-2909.121.3.417, PMID: 9136643

[ref4] Baer R. A. Smith G. T. Lykins E. Button D. Krietemeyer J. Sauer S.. (2008). Construct validity of the five facet mindfulness questionnaire in meditating and nonmeditating samples. Assessment 15, 329–342. doi: 10.1177/1073191107313003, PMID: 18310597

[ref5] Bar-Haim Y. Lamy D. Pergamin L. Bakermans-Kranenburg M. J. Van Ijzendoorn M. H. (2007). Threat-related attentional bias in anxious and nonanxious individuals: a meta-analytic study. Psychol. Bull. 133, 1–24. doi: 10.1037/0033-2909.133.1.1 17201568

[ref6] Baumeister R. F. (1984). Choking under pressure: self-consciousness and paradoxical effects of incentives on skillful performance. J. Pers. Soc. Psychol. 46, 610–620. doi: 10.1037//0022-3514.46.3.610, PMID: 6707866

[ref7] Baumeister R. F. Showers C. J. (1986). A review of paradoxical performance effects: choking under pressure in sports and mental tests. Eur. J. Soc. Psychol. 16, 361–383. doi: 10.1002/ejsp.2420160405

[ref8] Bedini L. A. Gladwell N. J. Dudley W. N. Clancy E. J. (2011). Mediation analysis of leisure, perceived stress, and quality of life in informal caregivers. J. Leis. Res. 43, 153–175. doi: 10.1080/00222216.2011.11950231

[ref9] Beilock S. L. Gray R. (2007). Why do athletes choke under pressure? Handbook of Sport Psychology, 425–444.

[ref10] Belinchon-deMiguel P. Ruisoto-Palomera P. Clemente-Suarez V. J. (2019). Psychophysiological stress response of a paralympic athlete during an ultra-endurance event. A case study. J. Med. Syst. 43:70. doi: 10.1007/s10916-019-1188-6, PMID: 30737600

[ref11] Bollen K. A. Stine R. (1990). Direct and indirect effects: classical and bootstrap estimates of variability. Sociol. Methodol. 20:115. doi: 10.2307/271084

[ref12] Brown K. W. Ryan R. M. (2003). The benefits of being present: mindfulness and its role in psychological well-being. J. Pers. Soc. Psychol. 84, 822–848. doi: 10.1037/0022-3514.84.4.822 12703651

[ref13] Burton A. Burgess C. Dean S. Koutsopoulou G. Z. Hugh-Jones S. (2017). How effective are mindfulness-based interventions for reducing stress among healthcare professionals? A systematic review and meta-analysis. Stress. Health 33, 3–13. doi: 10.1002/smi.2673, PMID: 26916333

[ref14] Butt J. Weinberg R. Culp B. (2010). Exploring mental toughness in NCAA athletes. J. Intercoll. Sport 3, 316–332. doi: 10.1123/jis.3.2.316

[ref15] Carlson L. E. Brown K. W. (2005). Validation of the mindful attention awareness scale in a cancer population. J. Psychosom. Res. 58, 29–33. doi: 10.1016/j.jpsychores.2004.04.366, PMID: 15771867

[ref16] Charoensukmongkol P. (2014). Benefits of mindfulness meditation on emotional intelligence, general self-efficacy, and perceived stress: evidence from Thailand. J. Spiritual. Ment. Health 16, 171–192. doi: 10.1080/19349637.2014.925364

[ref17] Chiesa A. Serretti A. (2010). A systematic review of neurobiological and clinical features of mindfulness meditations. Psychol. Med. 40, 1239–1252. doi: 10.1017/S0033291709991747, PMID: 19941676

[ref18] Cohen S. Kamarck T. Mermelstein R. (1983). A global measure of perceived stress. J. Health Soc. Behav. 24, 385–396. doi: 10.2307/2136404 6668417

[ref19] Connor K. M. Davidson J. R. (2003). Development of a new resilience scale: the Connor–Davidson resilience scale (CD-RISC). Depress. Anxiety 18, 76–82. doi: 10.1002/da.10113 12964174

[ref20] Eysenck M. W. Wilson M. R. (2016). “Sporting performance, pressure and cognition: introducing attentional control theory: sport” in An introduction to applied cognitive psychology. (Psychology Press), 341–362.

[ref21] Feldman G. Hayes A. Kumar S. Greeson J. Laurenceau J. P. (2007). Mindfulness and emotion regulation: the development and initial validation of the cognitive and affective mindfulness scale-revised (CAMS-R). J. Psychopathol. Behav. Assess. 29, 177–190. doi: 10.1007/s10862-006-9035-8

[ref22] Fornell C. Larcker D. F. (1981). Evaluating structural equation models with unobservable variables and measurement error. J. Mark. Res. 18, 39–50. doi: 10.1177/002224378101800104

[ref23] Gal E. Stefan S. Cristea I. A. (2021). The efficacy of mindfulness meditation apps in enhancing users’ well-being and mental health related outcomes: a meta-analysis of randomized controlled trials. J. Affect. Disord. 279, 131–142. doi: 10.1016/j.jad.2020.09.134, PMID: 33049431

[ref24] Geukes K. (2013). When the stakes are high: an application of the interactionist principle of trait activation to the prediction of performance under pressure.

[ref25] Ghorbani N. Krauss S. W. Watson P. LeBreton D. (2008). Relationship of perceived stress with depression: complete mediation by perceived control and anxiety in Iran and the United States. Int. J. Psychol. 43, 958–968. doi: 10.1080/00207590701295264, PMID: 22022839

[ref26] Gloria C. T. Steinhardt M. A. (2016). Relationships among positive emotions, coping, resilience and mental health. Stress. Health 32, 145, –156. doi: 10.1002/smi.2589 24962138

[ref27] Gucciardi D. F. Gordon S. Dimmock J. A. (2009). Advancing mental toughness research and theory using personal construct psychology. Int. Rev. Sport Exerc. Psychol. 2, 54–72. doi: 10.1080/17509840802705938

[ref28] Hao S. Hong W. Xu H. Zhou L. Xie Z. (2015). Relationship between resilience, stress and burnout among civil servants in Beijing, China: mediating and moderating effect analysis. Pers. Individ. Differ. 83, 65–71. doi: 10.1016/j.paid.2015.03.048

[ref29] Hill D. M. Hanton S. Matthews N. Fleming S. (2010). Choking in sport: a review. Int. Rev. Sport Exerc. Psychol. 3, 24–39. doi: 10.1080/17509840903301199

[ref30] Jones G. Hanton S. Connaughton D. (2002). What is this thing called mental toughness? An investigation of elite sport performers. J. Appl. Sport Psychol. 14, 205–218. doi: 10.1080/10413200290103509

[ref31] Kiken L. G. Lundberg K. B. Fredrickson B. L. (2017). Being present and enjoying it: dispositional mindfulness and savoring the moment are distinct, interactive predictors of positive emotions and psychological health. Mindfulness 8, 1280–1290. doi: 10.1007/s12671-017-0704-3, PMID: 29312472 PMC5755604

[ref32] Krane V. Joyce D. Rafeld J. (1995). Competitive anxiety, situation criticality, and softball performance. Sport Psychol. 8, 58–72. doi: 10.1123/tsp.8.1.58

[ref33] Kulsoom B. Afsar N. A. (2015). Stress, anxiety, and depression among medical students in a multiethnic setting. Neuropsychiatr. Dis. Treat. 11, 1713–1722. doi: 10.2147/ndt.S83577, PMID: 26213470 PMC4509544

[ref34] Lebares C. C. Guvva E. V. Ascher N. L. O’Sullivan P. S. Harris H. W. Epel E. S. (2018). Burnout and stress among US surgery residents: psychological distress and resilience. J. Am. Coll. Surg. 226, 80–90. doi: 10.1016/j.jamcollsurg.2017.10.010, PMID: 29107117

[ref35] Lehrer H. M. Steinhardt M. A. Dubois S. K. Laudenslager M. L. (2020). Perceived stress, psychological resilience, hair cortisol concentration, and metabolic syndrome severity: a moderated mediation model. Psychoneuroendocrinology 113:104510. doi: 10.1016/j.psyneuen.2019.104510, PMID: 31911349 PMC7769194

[ref36] Lemay V. Hoolahan J. Buchanan A. (2021). Impact of a yin yoga and meditation intervention on pharmacy faculty and student wellbeing. J. Am. Pharm. Assoc. 61, 703–708. doi: 10.1016/j.japh.2021.05.008, PMID: 34083148

[ref37] Lindsay M. Elsie D. (2016). Mindfulness: an effective prescription for depression and anxiety. J. Nurse Pract. 12, 403–409. doi: 10.1016/j.nurpra.2016.02.009

[ref38] Luthans F. (2002). The need for and meaning of positive organizational behavior. J. Organ. Behav. 23, 695–706. doi: 10.1002/job.165

[ref39] Madigan D. J. Nicholls A. R. (2017). Mental toughness and burnout in junior athletes: a longitudinal investigation. Psychol. Sport Exerc. 32, 138–142. doi: 10.1016/j.psychsport.2017.07.002

[ref40] Masrour M. J. Aerabsheybani H. Ramezani N. Aerabsheybani K. (2017). The effectiveness of mindfulness-based cognitive therapy (MBCT) in increasing infertile couples’ resilience and reducing anxiety, stress, and depression. Neuroquantology 15, 94–100. doi: 10.14704/nq.2017.15.3.1088

[ref41] Medina D. Y. (2016). Mindfulness matters: the influence of trait mindfulness on anxiety, executive control, and performance under pressure. California State University, Northridge.

[ref42] Mesagno C. Marchant D. Morris T. (2009). Alleviating choking: the sounds of distraction. J. Appl. Sport Psychol. 21, 131–147. doi: 10.1080/10413200902795091

[ref43] Mitchell A. E. P. (2021). Resilience and mindfulness in nurse training on an undergraduate curriculum. Perspect. Psychiatr. Care 57, 1474–1481. doi: 10.1111/ppc.12714 33355935

[ref44] Mossholder K. W. Bennett N. Kemery E. R. Wesolowski M. A. (1998). Relationships between bases of power and work reactions: the mediational role of procedural justice. J. Manage. 24, 533–552. doi: 10.1016/s0149-2063(99)80072-5

[ref45] Murray N. P. Janelle C. M. (2003). Anxiety and performance: a visual search examination of the processing efficiency theory. J. Sport Exerc. Psychol. 25, 171–187. doi: 10.1123/jsep.25.2.171

[ref46] Nicholls A. R. Polman R. C. J. Levy A. R. Hulleman J. (2012). An explanation for the fallacy of facilitative anxiety: stress, emotions, coping, and subjective performance in sport. Int. J. Sport Psychol. 43, 273–293.

[ref47] Papathomas A. (2007). Foundations of sport and exercise psychology. J. Sports Sci. 25:1625. doi: 10.1080/02640410701282405

[ref48] Podsakoff P. M. MacKenzie S. B. Podsakoff N. P. (2012). Sources of method bias in social science research and recommendations on how to control it. Annu. Rev. Psychol. 63, 539–569. doi: 10.1146/annurev-psych-120710-100452 21838546

[ref49] Scelzo A. Di Somma S. Antonini P. Montross L. P. Schork N. Brenner D.. (2018). Mixed-methods quantitative-qualitative study of 29 nonagenarians and centenarians in rural southern Italy: focus on positive psychological traits. Int. Psychogeriatr. 30, 31–38. doi: 10.1017/s1041610217002721, PMID: 29229012

[ref50] Sharma V. Sood A. Prasad K. Loehrer L. Schroeder D. Brent B. (2014). Bibliotherapy to decrease stress and anxiety and increase resilience and mindfulness: a pilot trial. Explore 10, 248–252. doi: 10.1016/j.explore.2014.04.002, PMID: 25037668

[ref51] Spielberger C. D. (1983). State-trait anxiety inventory for adults. PsycTests. doi: 10.1037/t06496-000

[ref52] Tang Y. Liu Y. Jing L. Wang H. Yang J. (2022). Mindfulness and regulatory emotional self-efficacy of injured athletes returning to sports: the mediating role of competitive state anxiety and athlete burnout. Int. J. Environ. Res. Public. Health 19:11702. doi: 10.3390/ijerph191811702, PMID: 36141969 PMC9517234

[ref53] Thurston I. B. Hardin R. Kamody R. C. Herbozo S. Kaufman C. (2018). The moderating role of resilience on the relationship between perceived stress and binge eating symptoms among young adult women. Eat. Behav. 29, 114–119. doi: 10.1016/j.eatbeh.2018.03.009, PMID: 29653301

[ref54] Trigueros R. Aguilar-Parra J. M. Alvarez J. F. Cangas A. J. Lopez-Liria R. (2020). The effect of motivation on the resilience and anxiety of the athlete. Rev. Int. Med. Cienc. Act. Fis. Deporte 20, 73–86. doi: 10.15366/rimcafd2020.77.005

[ref55] Tudose C. (2021). Anxiety, perceived stress, and resilience during the COVID-19 pandemic: population estimates of persons presenting to a general practitioner in Romania. Brain Sci. 11:1541. doi: 10.3390/brainsci11111541, PMID: 34827540 PMC8615933

[ref56] Wang J. (2002). Developing and testing an integrated model of choking in sport Victoria University.

[ref57] Wang J. Marchant D. Morris T. (2004). Coping style and susceptibility to choking. J. Sport Behav. 27, 75–92.

[ref58] Wathelet M. Duhem S. Vaiva G. Baubet T. Habran E. Veerapa E.. (2020). Factors associated with mental health disorders among university students in France confined during the COVID-19 pandemic. JAMA Netw. Open 3:e2025591. doi: 10.1001/jamanetworkopen.2020.25591, PMID: 33095252 PMC7584927

[ref59] White B. Driver S. Warren A.-M. (2008). Considering resilience in the rehabilitation of people with traumatic disabilities. Rehabil. Psychol. 53, 9–17. doi: 10.1037/0090-5550.53.1.9

[ref60] Wilson M. R. Wood G. Vine S. J. (2009). Anxiety, attentional control, and performance impairment in penalty kicks. J. Sport Exerc. Psychol. 31, 761–775. doi: 10.1123/jsep.31.6.761, PMID: 20384011

[ref61] Wu R. Jing L. Liu Y. Wang H. Yang J. (2022). Effects of physical activity on regulatory emotional self-efficacy, resilience, and emotional intelligence of nurses during the COVID-19 pandemic. Front. Psychol. 13:1059786. doi: 10.3389/fpsyg.2022.1059786, PMID: 36571052 PMC9780437

[ref62] Wulf G. (2013). Attentional focus and motor learning: a review of 15 years. Int. Rev. Sport Exerc. Psychol. 6, 77–104. doi: 10.1080/1750984X.2012.723728

[ref63] Ystgaard M. Tambs K. Dalgard O. S. (1999). Life stress, social support and psychological distress in late adolescence: a longitudinal study. Soc. Psychiatry Psychiatr. Epidemiol. 34, 12–19. doi: 10.1007/s001270050106, PMID: 10073116

